# Effectiveness of XBB.1.5 Vaccines Against Symptomatic SARS‐CoV‐2 Infection in Older Adults During the JN.1 Lineage‐Predominant Period, European VEBIS Primary Care Multicentre Study, 20 November 2023–1 March 2024

**DOI:** 10.1111/irv.70009

**Published:** 2024-11-10

**Authors:** Lore Merdrignac, Charlotte Laniece Delaunay, Nuno Verdasca, Lorena Vega‐Piris, Joan O'Donnell, Noémie Sève, Camino Trobajo‐Sanmartín, Silke Buda, Mariëtte Hooiveld, Ana Paula Rodrigues, Gergő Túri, Neus Latorre‐Margalef, Ivan Mlinarić, Mihaela Lazar, Marine Maurel, Daniel Castrillejo, Charlene Bennett, Marie‐Anne Rameix‐Welti, Iván Martínez‐Baz, Ralf Dürrwald, Adam Meijer, Aryse Melo, Beatrix Oroszi, Tove Samuelsson Hagey, Sanja Kurečić Filipović, Frederika Dijkstra, Veronica Gomez, Sabrina Bacci, Marlena Kaczmarek, Esther Kissling, the VEBIS Primary Care Vaccine Effectiveness Group

**Affiliations:** ^1^ Epidemiology Department Epiconcept Paris France; ^2^ National Reference Laboratory for Influenza Virus and Other Respiratory Viruses, Infectious Disease Department National Institute of Health Doutor Ricardo Jorge Lisbon Portugal; ^3^ National Centre of Epidemiology, CIBERESP Carlos III Health Institute Madrid Spain; ^4^ Health Service Executive‐Health Protection Surveillance Centre Dublin Ireland; ^5^ Sorbonne Université, INSERM, Institut Pierre Louis d'Epidémiologie et de Santé Publique, IPLESP Paris France; ^6^ Instituto de Salud Pública de Navarra ‐ IdiSNA – CIBERESP Pamplona Spain; ^7^ Department of Infectious Disease Epidemiology Robert Koch Institute Berlin Germany; ^8^ Nivel Utrecht the Netherlands; ^9^ Epidemiology Department National Institute of Health Doutor Ricardo Jorge Lisbon Portugal; ^10^ National Laboratory for Health Security, Epidemiology and Surveillance Centre Semmelweis University Budapest Hungary; ^11^ Department of microbiology Public Health Agency of Sweden Stockholm Sweden; ^12^ Division for Epidemiology of Communicable Diseases Croatian Institute of Public Health Zagreb Croatia; ^13^ National Influenza Centre Cantacuzino National Military Medical Institute for Research and Development Bucharest Romania; ^14^ Servicio de Vigilancia Epidemiológica, Dirección General de Salud Pública Consejería de Políticas Sociales y Salud Pública Melilla Spain; ^15^ National Virus Reference Laboratory University College Dublin Dublin Ireland; ^16^ Centre National de Référence Virus des Infections Respiratoire (CNR VIR) Institut Pasteur Université Paris Cité Paris France; ^17^ Molecular Mechanisms of Multiplication of Pneumovirus Unit, UMR 1173 (2I) Institut Pasteur, Université Paris‐Saclay, Université de Versailles St. Quentin, INSERM, Université Paris Cité Paris France; ^18^ National Reference Centre for Influenza Robert Koch Institute Berlin Germany; ^19^ National Institute for Public Health and the Environment Centre for Infectious Diseases Control Bilthoven the Netherlands; ^20^ Vaccine Preventable Diseases and Immunisation Section European Centre for Disease Prevention and Control Stockholm Sweden

**Keywords:** case–control study, COVID‐19, multi‐country study, primary care, SARS‐CoV‐2, test‐negative design, vaccine effectiveness

## Abstract

We estimated XBB.1.5 vaccine effectiveness (VE) against symptomatic SARS‐CoV‐2 infection among adults aged ≥ 65 years during the 2023/2024 JN.1 lineage‐predominant period in a European multi‐country test‐negative case–control study at primary care level. We estimated VE adjusted by study site, age, sex, chronic conditions and onset date. We included 220 cases and 1733 controls. The VE was 48% (95% CI: 12–71), 23% (95% CI: −11–48) and 5% (95% CI: −92–56) among those with symptom onset 1–5, 6–11, and ≥ 12 weeks after vaccination, respectively. XBB.1.5 vaccine provided short and moderate protection against JN.1 symptomatic infection.

## 1 Introduction

During the 2023/2024 season, the SARS‐CoV‐2 JN.1 lineage, a sublineage of BA.2.86, rapidly displaced XBB lineages. As of 30 April 2024, > 90% of SARS‐CoV‐2 viruses sequenced globally were JN.1 or its descendants [1]. JN.1 harbours the L455S substitution in the receptor binding site in the spike protein, which may be associated with immune escape [2].

In the autumn/winter 2023/2024 COVID‐19 vaccination campaigns in Europe, the XBB.1.5 vaccine was recommended in most countries, comprising over 97% of vaccines administered [3]. Older adults were a priority group in these campaigns, as well as those with medical risk conditions and healthcare workers. In certain European countries, the XBB.1.5 vaccines have also been used for spring 2024 campaigns.

Regular monitoring of COVID‐19 vaccine effectiveness (VE) is important to understand the performance of the vaccine in light of evolving variants and underlying population characteristics, including by time since vaccination.

The VEBIS (Vaccine Effectiveness, Burden and Impact Studies) project is a European multicentre test‐negative case–control study. We estimated autumn/winter 2023/2024 COVID‐19 VE against medically attended symptomatic SARS‐CoV‐2 infection in older Europeans at primary care level during a period of JN.1‐predominance.

## 2 Methods

### 2.1 Study Design, Data Collection and Key Definitions

Eleven study sites in 10 European countries participated in the VEBIS primary care study: Croatia, France, Germany, Hungary, Ireland, the Netherlands, Portugal, Romania, Spain (national), Spain (Navarre region) and Sweden.

Methods have been described elsewhere [4, 5]. Briefly, participating physicians selected all or a systematic sample of patients with acute respiratory infection (ARI) for swabbing and inclusion in the study. Samples were tested by RT‐PCR for SARS‐CoV‐2 and influenza. Those testing positive for SARS‐CoV‐2 were cases, and those testing negative were controls. Demographic (age and sex) and clinical (symptoms, dates of symptom onset and swabbing) information were collected. COVID‐19 vaccination information was collected through vaccine registry linkage, electronic health record look‐up and self‐report. Site‐specific information on patient recruitment, case definitions, 2023/2024 COVID‐19 vaccination campaign information and data sources are specified in Table 1. We defined a patient as vaccinated if they received 2023/2024 COVID‐19 vaccine on or after the start of their country/region‐specific autumn 2023/2024 vaccination campaign and ≥ 7 days before symptom onset. To estimate VE, we defined the reference group of ‘unvaccinated’ individuals as those patients who did not receive 2023/2024 COVID‐19 vaccine either within the study period before recruitment or during the 180 days prior to the start of the 2023/2024 COVID‐19 vaccination campaign. Study sites sequenced all or a random sample of SARS‐CoV‐2 viruses below a specified cycle threshold (Ct) value.

**TABLE 1:** Autumn/winter 2023/2024 national COVID‐19 vaccination campaign information and study site‐specific information on case definitions, vaccination data sources and JN.1 lineage‐predominant period start, VEBIS primary care study (eight study sites^a^), Europe, 20 November 2023–1 March 2024.

Study site	Start of autumn 2023 national COVID‐19 vaccination campaigns	Recommendation for COVID‐19 vaccination for all adults over this age (years)	Case definition used for recruitment of patients^b^	Data source for COVID‐19 vaccination information	JN.1 lineage‐predominant period start (≥ 60%)^c^
France	2 October 2023	65	Sentinelles ARI	GP interview (self‐report)	11 December 2023
Germany	18 September 2023	60	ARI	Medical records, patient's certificate of vaccination, GP interview (self‐report)	18 December 2023
Hungary	1 October 2023	60	EU‐ARI	Primarily from the National Immunisation Registry (83.5%), or from GP records or self‐report	8 January 2024
Ireland	2 October 2023	50	EU‐ARI	Data linkage to vaccine registry	11 December 2023
The Netherlands	2 October 2023	60	EU‐ILI or EU‐ARI	GP interview (self‐report)	11 December 2023
Portugal	29 September 2023	60	EU‐ARI	Vaccine registry look‐up by GPs	20 November 2023
Spain, national	25 September 2023	60	EU‐ARI	Data linkage to vaccine registry	4 December 2023
Spain, Navarre region	16 October 2023	60	EU‐ILI	Data linkage to vaccine registry	4 December 2023

Abbreviations: ARI, acute respiratory infection; EU, European Union; ILI, influenza‐like illness; VEBIS, Vaccine Effectiveness, Burden and Impact Studies.

^a^ Eight study sites were included in the 2023/2024 COVID‐19 VE analysis in the JN.1 lineage‐predominant period (providing ≥ 10 COVID‐19 cases): France, Germany, Hungary, Ireland, the Netherlands, Portugal, Spain (national) and Spain (Navarree region).

^b^ EU‐ARI: sudden onset of symptoms and at least one of four respiratory symptoms (cough, sore throat, shortness of breath, coryza) and a clinician's judgement that the illness is due to an infection; EU‐ILI: sudden onset of symptoms and at least one of four systemic symptoms (fever or feverishness, malaise, headache or myalgia) and at least one of three respiratory symptoms (cough, sore throat, or shortness of breath); Sentinelles ARI: sudden onset of fever (or feverishness) and respiratory signs; the ARI case definition in Germany includes patients with at least one of the following four symptoms: fever, cough, coryza or sore throat.

^c^ Due to low samples sequenced by week, GISAID data for Hungary were imputed using data from the seven countries sharing a border (Austria, Croatia, Romania, Serbia, Slovenia, Slovakia and the Ukraine).

For analysis, we restricted to those patients swabbed within 10 days of symptom onset, meeting the EU‐ARI case definition (sudden onset of symptoms and at least one of four respiratory symptoms [cough, sore throat, shortness of breath and coryza]), with complete and consistent key variable information, eligible for XBB.1.5 vaccination in the 2023/2024 season. We excluded patients with non‐JN.1 or descendant characterised viruses and excluded study sites with fewer than 10 cases or controls each.

We determined the start of the JN.1 lineage‐predominant week from the week in which ≥ 60% of sequenced viruses were JN.1 viruses, by country, as reported by GISAID [6]. If countries sequenced a low number of viruses on a weekly basis, sequences in the neighbouring countries were used as a proxy (Table 1). The end of the study period was at the time of last study site data extraction, up to 1 March 2024.

### 2.2 Statistical and VE Analyses

We pooled individual patient data from each study site and described the data according to baseline characteristics of cases and controls. In a primary analysis, we restricted to those aged 65 years and older in order to better compare to the literature and in a secondary analysis to those who were part of the age‐specific target group for vaccination in each country, with ages ranging between 50 and 65 years and older (Table 1).

Using logistic regression, we estimated VE as (1 − odds ratio) * 100, with study site as fixed effects and adjusting for a priori confounders of age, sex, onset date and presence of at least one of four commonly collected chronic conditions. We used the Akaike information criterion to select the best functional form of the continuous variables age and onset date, modelled as either a restricted cubic spline with three to five knots, categorical or continuous variables. We estimate VE overall and by time since vaccination using cut‐offs of 1–5, 6–11 and ≥ 12 weeks.

All analyses were performed in R software Version 4.3.2 (R Project for Statistical Computing).

Using the 10 events per parameter rule of thumb, we re‐analysed the data using Firth's penalised logistic regression if the number of parameters in the model exceed the number of cases (or controls) divided by 10 [7]. If the VE estimates of standard and Firth's logistic regression differed by > 10%, we excluded the estimates, assuming bias due to overfitting.

### 2.3 Sensitivity Analyses

We estimated VE defining a person as vaccinated if they received vaccination 14 days or more before symptom onset, rather than 7 days, in order to better compare to the literature. We also used monthly cut‐offs for time since vaccination: 7–29, 30–59, 60–89 and ≥ 90 days.

## 3 Results

**FIGURE 1:**
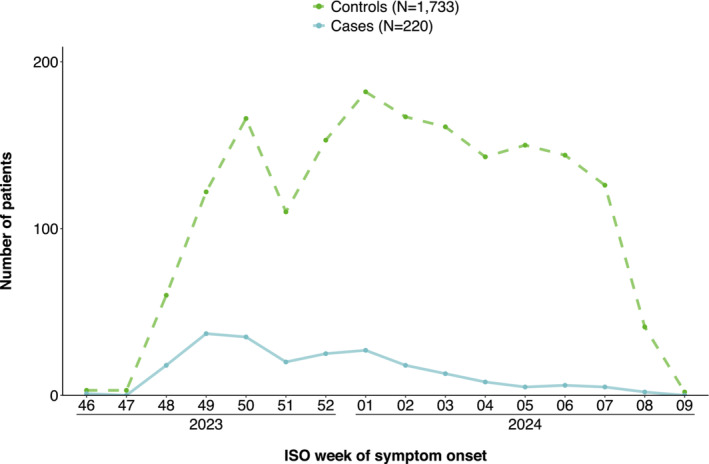
Patients included in the VEBIS primary care study by COVID‐19 case status and ISO week of symptom onset, Europe, 20 November 2023–1 March 2024 (*n* = 1953). ISO, International Organisation for Standardisation; VEBIS, Vaccine Effectiveness, Burden and Impact Studies.

We included 1953 patients aged 65 years and older, of which 220 were cases and 1733 controls. Our study period ranged from 20 November 2023 to 1 March 2024 (Figure 1). The median age of both cases and controls was 74 years (Table 2). Among cases, 34% (75/220) were vaccinated against COVID‐19 in the 2023/2024 autumn/winter campaign vs. 43% (753/1733) of controls (Table 2). Vaccine brand was known among 562 of 828 vaccination patients (68%), of these 561 (100%) were vaccinated with Comirnaty and one with Spikevax.

**TABLE 2:** Baseline characteristics of patients included in the VEBIS primary care study by COVID‐19 case status among adults aged ≥ 65 years, during the JN.1 lineage‐predominant period, Europe, 20 November 2023–1 March 2024.

Variables	Number of COVID‐19 cases (%) *n* = 220	Number of test‐negative controls (%) *n* = 1733
Median age (IQR)	74 (69–80)	74 (69–80)
Age groups (years)		
65–69	57/220 (26)	471/1733 (27)
70–79	107/220 (49)	823/1733 (47)
≥ 80	56/220 (25)	439/1733 (25)
Female	140/220 (64)	1005/1733 (58)
Presence of four commonly collected chronic conditions^a^	159/220 (72)	1248/1733 (72)
Received COVID‐19 vaccination as part of the autumn/winter 2023/2024 campaign	75/220 (34)	753/1733 (43)
Number of COVID‐19 doses received at time of autumn/winter 2023/2024 campaign^b^		
1	0/75 (0)	1/747 (0)
2	0/75 (0)	2/747 (0)
3	2/75 (3)	15/747 (2)
4	5/75 (7)	95/747 (13)
5	59/75 (79)	542/747 (73)
6	6/75 (8)	48/747 (6)
7	3/75 (4)	43/747 (6)
8	0/75 (0)	1/747 (0)
Missing	0	6
Median number of days between last COVID‐19 vaccination received in the autumn/winter 2023/2024 campaign and symptom onset (IQR) ^b^	54 (45–77)	67 (47–87)
Influenza virus RT‐PCR positive	11/220 (5)	329/1721 (19)
Missing	0	12

Abbreviations: IQR, interquartile range; VEBIS, Vaccine Effectiveness, Burden and Impact Studies.

^a^ Diabetes, heart disease, chronic lung disease, and immunodeficiency.

^b^ Out of all those vaccinated in the autumn/winter 2023/2024 COVID‐19 vaccination campaigns.

The VE among those aged ≥ 65 years was 28% (95% CI: 2–48) overall and 48% (95% CI: 12–71), 23% (95% CI: −11–48) and 5% (95% CI: −92–56) at 1–5, 6–11 and ≥ 12 weeks after vaccination, respectively (Table 3).

**TABLE 3:** Pooled vaccine effectiveness against medically attended SARS‐CoV‐2 infection among older adults during the JN.1 lineage‐predominant period, VEBIS primary care study, Europe, 20 November 2023–1 March 2024.

Analysis	TSV	Cases	Median TSV in days among cases (IQR)	Controls	Median TSV among controls (IQR)	VE (95% CI)
Adults aged ≥ 65 years	Unvaccinated	145	—	980	—	—
	Overall	75	54 (45–77)	753	67 (47–87)	28 (2–48)
	1–5 weeks	18	25.5 (20.25–38)	143	29 (20–36)	48 (12–71)
	6–11 weeks	46	55.5 (49–72.25)	397	63 (53–74)	23 (−11–48)
	12–20 weeks	11	96 (90.5–106)	213	99 (90–110)	5 (−92–56)
Older adults part of the age‐specific COVID‐19 vaccination campaign^a^	Unvaccinated	219		1574		
	Overall	92	55 (46–76)	920	66 (47–86)	26 (3–44)
	1–5 weeks	20	24 (20–38)	178	29 (20–36)	51 (20–71)
	6–11 weeks	59	56 (50.5–72)	491	63 (53–74)	17 (−15–40)
	12–20 weeks	13	96 (89–111)	251	99 (90–110)	10 (−64–54)

Abbreviations: CI, confidence interval; TSV, time since vaccination; VE, vaccine effectiveness; VEBIS, Vaccine Effectiveness, Burden and Impact Studies.

^a^ The age‐specific recommendation for 2023/2024 COVID‐19 vaccination was among adults aged ≥ 50, ≥ 60 or ≥ 65 years, depending on study site (see Table 1).

In our secondary analysis among older adults who were part of the countries' age‐specific target group for vaccination, there was overlap with the ≥ 65‐year‐old age group analysis, with 71% of cases and 69% of controls aged ≥ 65 years. The VE point estimates differed by ≤ 6%, depending on analysis (Table 3).

In the sensitivity analysis with a patient considered as ‘immunised’ after 14 days post‐vaccination, instead of 7 days, all VE point estimates differed by < 2% (Table S1). When using monthly cut‐offs for time since vaccination, the VE point estimates were 40% (95% CI: −15–72), 28% (95% CI: −9–53), 29% (95% CI: −15–58) and 5% (95% CI: −110–61) at 7–29, 30–59, 60–89 and ≥ 90 days after vaccination (Table S2).

## 4 Discussion

Our results indicate that XBB.1.5 VE against JN.1 symptomatic infection among adults aged ≥ 65 years medically attended at primary care level was initially moderate at 48% 1–5 weeks after vaccination, but declined quickly to 23% within 6–11 weeks, with little effect from 12 weeks after vaccination onwards, although precision was low, particularly from 12 weeks onwards.

We are not aware of other outpatient‐based studies among older adults estimating symptomatic COVID‐19 VE due to JN.1. Among studies of persons aged ≥ 18 years, Caffrey et al. report a VE of 31% within 60 days of vaccination and 20% within 61–133 days [8]. Link‐Gelles et al. report a VE of 49% among a study population with median delay of 80 days since vaccination [9]. Our results suggest a potentially lower VE with more time since vaccination than those studies, although comparison is difficult without similar groupings for time since vaccination in the other studies. Additionally, due to low sample size and precision among those with 12 weeks since vaccination, it is difficult to conclude from our study if the decline in VE by time since vaccination is moderate or rapid from its peak.

We previously reported within the VEBIS primary care study a VE of 45% (95% CI: 26–60) within 1–5 weeks of vaccination among adults in the older age target group in the early 2023/2024 season (September 2023 to January 2024), when the majority of viruses were likely to be XBB and its descendants [10]. When linking epidemiological and sequencing data, the XBB lineage‐specific VE in the same study and age group was 63% (95% CI: −2–90) within 1–5 weeks of vaccination. Comparison to the current VE of 51% (95% CI: 20–71) against JN.1 within the same population and time since vaccination suggests no difference in VE or at most a modestly higher VE against XBB than against JN.1. Neutralisation studies indicate that JN.1 has more immune escape compared to XBB, although the drop in neutralisation titres are moderate [11]. VE in other studies suggest potentially greater differences in VE between XBB and JN.1 than we observe [9, 12, 13]. More studies on VE against symptomatic infection in the XBB and JN.1 lineage‐predominant periods among older adults are needed to help understand if our results are due to small sample size and/or differing population characteristics or if XBB.1.5 vaccine may work similarly against XBB and JN.1 lineages within a very short time after vaccination.

As with all observational studies, our study is subject to residual and unmeasured confounding. Additionally, the low sample size of cases in the JN.1 period limits the precision around our estimates. VE among those vaccinated within 12–20 weeks of symptom onset was particularly imprecise, and inferences should be made with caution. Due to low numbers and in particular low vaccine coverage among the younger target group for vaccination (< 50–64 years, depending on country‐specific recommendations), we were unable to provide estimates in this group within the JN.1 period. Our previous work suggests VE may be higher in this younger target group [10]. The Spanish study site dominated in our study (73%, data not shown). Although we are not aware of any differences in terms of vaccine brands or schedules used in Spain compared to other countries, we cannot exclude heterogeneity among countries in terms of previous SARS‐CoV‐2 infection, which may result in heterogeneity in VE. We determined the JN.1 lineage‐predominant period using external surveillance data, as sample size was too small for an analysis using within‐study sequencing data. Using a 60% cut‐off may have resulted in contamination of our estimates with non‐JN.1 viruses, although mainly in the early study period, as the proportion JN.1 viruses increased rapidly.

A strength of our study is to be able to provide COVID‐19 VE results in the outpatient setting, where there are limited studies. Our long‐standing platform can provide VE results across seasons, which is useful for comparison. Ongoing monitoring of VE is key in the context of the WHO recommendation of JN.1 vaccines in the autumn/winter 2024/2025 vaccination campaigns [14] and the diversification of JN.1 viruses into sublineages.

In conclusion, our results suggest XBB.1.5 vaccines provide short and moderate protection against symptomatic infection with JN.1 lineages among older patients, similar to the VE against predominantly XBB lineages earlier in the season. Careful timing of COVID‐19 vaccination campaigns is needed to ensure optimal protection of vulnerable populations.

## Author Contributions

**Lore Merdrignac:** conceptualisation, formal analysis, visualisation, methodology, writing – review and editing. **Charlotte Laniece Delaunay:** conceptualisation, formal analysis, methodology. **Nuno Verdasca:** conceptualisation, data curation, formal analysis, writing – review and editing. **Lorena Vega‐Piris:** conceptualisation, investigation, methodology, writing – review and editing. **Joan O'Donnell:** conceptualisation, investigation, methodology, writing – review and editing. **Noémie Sève:** conceptualisation, investigation, methodology, writing – review and editing. **Camino Trobajo‐Sanmartín:** conceptualisation, investigation, methodology, writing – review and editing. **Silke Buda:** conceptualisation, investigation, methodology, writing – review and editing. **Mariëtte Hooiveld:** conceptualisation, investigation, methodology, writing – review and editing. **Ana Paula Rodrigues:** conceptualisation, investigation, methodology, writing – review and editing**. Gergő Túri:** conceptualisation, investigation, methodology, writing – review and editing. **Neus Latorre‐Margalef:** conceptualisation, investigation, methodology, writing – review and editing. **Ivan Mlinarić:** conceptualisation, investigation, methodology, writing – review and editing. **Mihaela Lazar:** conceptualisation, investigation, methodology, writing – review and editing. **Marine Maurel:** conceptualisation, methodology, visualisation, data curation, writing – review and editing. **Daniel Castrillejo:** conceptualisation, investigation, methodology, writing – review and editing. **Charlene Bennett:** investigation, methodology, writing – review and editing. **Marie‐Anne Rameix‐Welti:** conceptualisation, investigation, methodology, writing – review and editing. **Iván Martínez‐Baz:** conceptualisation, investigation, methodology, writing – review and editing. **Ralf Dürrwald:** conceptualisation, investigation, methodology, writing – review and editing. **Adam Meijer:** conceptualisation, investigation, methodology, writing – review and editing. **Aryse Melo:** conceptualisation, investigation, methodology, writing – review and editing. **Beatrix Oroszi:** conceptualisation, investigation, methodology, writing – review and editing. **Tove Samuelsson Hagey:** conceptualisation, investigation, methodology, writing – review and editing. **Sanja Kurečić Filipović:** conceptualisation, investigation, methodology, writing – review and editing. **Frederika Dijkstra:** conceptualisation, investigation, methodology, writing – review and editing. **Veronica Gomez:** conceptualisation; investigation, methodology, writing – review and editing. **Sabrina Bacci:** conceptualisation, project administration, funding acquisition, writing – review and editing. **Marlena Kaczmarek:** conceptualisation, project administration, methodology, funding acquisition, writing – review and editing. **Esther Kissling:** conceptualisation; writing – original draft, methodology, project administration, writing – review and editing. **The VEBIS Primary Care Vaccine Effectiveness Group:** investigation, data curation.

## Ethics Statement

Official ethical approval was not required in Spain or the Netherlands, as this study was classified as routine care/surveillance. Other study sites received local ethical approval from a national or regional review board: Croatia: (class 030‐02/23‐01/1); France: 471393; Germany: EA2/126/11; Hungary: IV/1885‐5/2021/EKU; Ireland: ICGP2019.4.04; Portugal: no registration number given; Romania: CE199/2022; Sweden: 2006/1040‐31/2 revised Drn 2021‐02791.

## Consent

Patient consent was not required in Ireland or Spain. Verbal consent was required for all other study sites, with the exception of Germany and Hungary, where written consent was required.

## Conflicts of Interest

The authors declare no conflicts of interest.

## Supporting information


**Table S1** Pooled vaccine effectiveness against medically attended SARS‐CoV‐2 infection among older adults during the JN.1 lineage‐predominant period, using 14 instead of 7 days as immunisation definition^a^, VEBIS primary care study, Europe, 20 November 2023–1 March 2024.
**Table S2.** Pooled vaccine effectiveness against medically attended SARS‐CoV‐2 infection among older adults during the JN.1 lineage‐predominant period, by monthly time since vaccination cutoffs, VEBIS primary care study, Europe, 20 November 2023–1 March 2024.

## Data Availability

The data that support the findings of this study are available upon reasonable request.
