# Supplementary material for: Effectiveness of XBB.1.5 Vaccines Against Symptomatic SARS‐CoV‐2 Infection in Older Adults During the JN.1 Lineage‐Predominant Period, European VEBIS Primary Care Multicentre Study, 20 November 2023–1 March 2024
Source: Influenza Other Respir Viruses. 2024 Nov 10;18(11):e70009. doi: 10.1111/irv.70009 (PMC11551475; doi:10.1111/irv.70009)
Supplement: Supplementary file 1 — Table S1 Pooled vaccine effectiveness against medically attended SARS‐CoV‐2 infection among older adults during the JN.1 lineage‐predominant period, using 14 instead of 7 days as immunisation definitiona, VEBIS primary care study, Europe, 20 November 2023–1 March 2024. Table S2. Pooled vaccine effectiveness against medically attended SARS‐CoV‐2 infection among older adults during the JN.1 lineage‐predominant period, by monthly time since vaccination cutoffs, VEBIS primary care study, Europe, 20 November 2023–1 March 2024. [file IRV-18-e70009-s001.docx]

**Supplement**

**Table S1.** Pooled vaccine effectiveness against medically attended SARS-CoV-2 infection among older adults during the JN.1 lineage-predominant period, using 14 instead of 7 days as immunisation definition^a^, VEBIS primary care study, Europe, 20 November 2023–1 March 2024

| **Analysis** | **Time since vaccination** |  |  | **Cases** |  | **Controls** | **VE (95% CI)** |
| --- | --- | --- | --- | --- | --- | --- | --- |
| Adults aged ≥65 years | Unvaccinated |  |  | 145 |  | 980 | - |
|  | Overall |  |  | 73 |  | 738 | 27 (1–47) |
|  | 1–5 weeks |  |  | 16 |  | 128 | 48 (10–72) |
|  | 6–11 weeks |  |  | 46 |  | 397 | 23 (-11–48) |
|  | 12–20 weeks |  |  | 11 |  | 213 | 5 (-92–56) |
| Older adults part of the age-specific COVID-19 vaccination campaign^b^ | Unvaccinated |  |  | 219 |  | 1574 | - |
|  | Overall |  |  | 90 |  | 896 | 24 (0–43) |
|  | 1–5 weeks |  |  | 18 |  | 154 | 49 (15–71) |
|  | 6–11 weeks |  |  | 59 |  | 491 | 17 (-15–40) |
|  | 12–20 weeks |  |  | 13 |  | 251 | 10 (-64–54) |
| Abbreviations: VEBIS, Vaccine Effectiveness, Burden and Impact Studies; N, number; VE, vaccine effectiveness; CI, confidence interval.  ^a^ This analysis results also in the study period starting 14 days after the COVID-19 vaccination campaigns, by country, not 7 days, as in the main analysis.  ^b^ The age-specific recommendation for 2023/24 COVID-19 vaccination was among adults aged ≥50, ≥60 or ≥65 years, depending on study site (see Table 1).  **Table S2.** Pooled vaccine effectiveness against medically attended SARS-CoV-2 infection among older adults during the JN.1 lineage-predominant period, by monthly time since vaccination cutoffs, VEBIS primary care study, Europe, 20 November 2023–1 March 2024   \| **Analysis** \| **Time since vaccination** \|  \|  \| **Cases** \|  \| **Controls** \| **VE (95% CI)** \| \| --- \| --- \| --- \| --- \| --- \| --- \| --- \| --- \| \| Adults aged ≥65 years \| Unvaccinated \|  \|  \| 145 \|  \| 980 \| - \| \| Overall \|  \|  \| 75 \|  \| 753 \| 28 (2–48) \| \| 7–29 days \|  \|  \| 11 \|  \| 72 \| 40 (-15–72) \| \| 30–59 days \|  \|  \| 34 \|  \| 232 \| 28 (-9–53) \| \| 60–89 days \|  \|  \| 22 \|  \| 282 \| 29 (-15–58) \| \| 90+ days \|  \|  \| 8 \|  \| 167 \| 5 (-110–61) \| \| Older adults part of the age-specific COVID-19 vaccination campaign^a^ \| Unvaccinated \|  \|  \| 219 \|  \| 1574 \| - \| \| Overall \|  \|  \| 92 \|  \| 920 \| 26 (3–44) \| \| 7–29 days \|  \|  \| 13 \|  \| 91 \| 38 (-13–68) \| \| 30–59 days \|  \|  \| 41 \|  \| 289 \| 27 (-5–50) \| \| 60–89 days \|  \|  \| 29 \|  \| 342 \| 23 (-17–51) \| \| 90+ days \|  \|  \| 9 \|  \| 198 \| 11 (-79–61) \| \| Abbreviations: VEBIS, Vaccine Effectiveness, Burden and Impact Studies; N, number; VE, vaccine effectiveness; CI, confidence interval. \| \| \| \| \| \| \| \|   ^a^ The age-specific recommendation for 2023/24 COVID-19 vaccination was among adults aged ≥50, ≥60 or ≥65 years, depending on study site (see Table 1). | | | | | | | |
